# Sputum Microbiota Associated with New, Recurrent and Treatment Failure Tuberculosis

**DOI:** 10.1371/journal.pone.0083445

**Published:** 2013-12-13

**Authors:** Jing Wu, Wei Liu, Lei He, Fuli Huang, Jiazhen Chen, Peng Cui, Yaojie Shen, Jing Zhao, Wenjie Wang, Yan Zhang, Min Zhu, Wenhong Zhang, Ying Zhang

**Affiliations:** 1 Department of Infectious Diseases, Huashan Hospital, Fudan University, Shanghai, China; 2 Hangzhou Center for Disease Control and Prevention, Zhejiang, China; 3 Department of Tuberculosis Branch, Hangzhou Red Cross Hospital, Zhejiang, China; 4 Institute of Biomedical Sciences, Fudan University, Shanghai, China; 5 MOH and MOE Key Laboratory of Medical Molecular Virology, Shanghai Medical College, Fudan University, Shanghai, China; 6 Department of Molecular Microbiology and Immunology, Bloomberg School of Public Health, Johns Hopkins University, Baltimore, Maryland, United States of America; Institut de Pharmacologie et de Biologie Structurale, France

## Abstract

Microbiota have recently been shown to be associated with many disease conditions. However, the microbiota associated with tuberculosis (TB) infection, recurrence and treatment outcome have not been systematically characterized. Here, we used high throughput 16S RNA sequencing to analyze the sputum microbiota associated with *Mycobacterium tuberculosis* infection and also to identify the microorganisms associated with different outcomes of TB treatment. We recruited 25 new TB patients, 30 recurrent TB patients and 20 TB patients with treatment failure, as well as 20 healthy controls. *Streptococcus*, *Gramulicatella* and *Pseudomonas* were more abundant in TB patients while *Prevotella*, *Leptotrichia*, *Treponema*, *Catonella* and *Coprococcus* were less abundant in TB patients than in the healthy controls. We found reduced frequency and abundance of some genera such as *Bulleidia* and *Atopobium* in recurrent TB patients compared with those in new TB patients. In addition, the ratio of *Pseudomonas* / *Mycobacterium* in recurrent TB was higher than that in new TB while the ratio of *Treponema* / *Mycobacterium* in recurrent TB was lower than that in new TB, indicating that disruption of these bacteria may be a risk factor of TB recurrence. Furthermore, *Pseudomonas* was more abundant and more frequently present in treatment failure patients than in cured new patients, and the ratio of *Pseudomonas* / *Mycobacterium* in treatment failure was higher than that in new TB. Our data suggest that the presence of certain bacteria and the disorder of lung microbiota may be associated with not only onset of TB but also its recurrence and treatment failure. These findings indicate that lung microbiota may play a role in pathogenesis and treatment outcome of TB and may need to be taken into consideration for improved treatment and control of TB in the future.

## Introduction

Tuberculosis caused by *Mycobacterium tuberculosis* (*Mtb*) remains a leading infectious disease worldwide despite availability of chemotherapy and BCG vaccine. Each year there are 9 million new cases and close to 2 million deaths (WHO). One third of the world population, about 2 billion people, is latently infected with *Mtb*. In people with latent tuberculosis infection, the risk of becoming active tuberculosis is approximately 5% - 10% during the life time. When the immune system is compromised, such as co-infections with HIV, malnutrition and aging, latent infections can reactivate and develop into active disease [1]. Although TB can be cured with a cure rate of 85-95%, some patients are not cured and even cured patients can have relapse. While many factors such as inadequate patient compliance and drug resistance are involved, they do not appear to explain all the treatment failures or relapse. Circumstantial observations seem to suggest co-infection or prior infection with other pathogens might play a role in pathogenesis and onset of TB. It is possible that resident lung microbial community or microbiota may play a role in the disease reactivation and treatment outcome. 

Recently, high-throughput sequencing technologies have been applied to characterize human microbiota [2] in various body habitats such as gut [3], oral cavity [4,5], vagina [6] and respiratory tract [7]. Human lung microbiota was identified by 16S rRNA sequencing [8], demonstrating that the lung is not sterile contrary to previous belief. Besides, complicated bacterial communities associated with respiratory diseases were also identified by analysis of the composition of bacteria by deep sequencing in sputum samples of cystic fibrosis patients, whereas the low-prevalence or fastidious bacteria were not detected by standard clinical culture methods [9,10]. Deep sequencing also shows higher sensitivity than other detection system like Vitek 2 Compact system in detecting lower respiratory tract infections [11]. The number of studies investigating the role of sputum microbiome in tuberculosis is limited. While this work was ongoing, two recent studies characterizing the composition and diversity of sputum microbiota in TB patients compared with healthy controls were reported [12,13]. However, the possible role of microbiota in TB recurrence and treatment outcome has not been investigated. 

In this study, we used multiplexed barcoded 16S rRNA sequencing to characterize the sputum microbiota in new pulmonary TB, recurrent TB, and treatment failure TB patients, in order to shed light on the lung microbiota that may be associated with outcomes of TB infection and treatments. 

## Materials and Methods

### Ethics Statement

This study was approved by the ethics review committee of Huashan Hospital, School of Medicine, Fudan University. Written informed consent was obtained from either the participants or their guardians. If the participants were under 18 years of age, their parents provided written informed consent on their behalf. Sputum or throat swab samples were collected and participant information was collected including age, sex, underlying diseases, clinical manifestations, and laboratory and radiological findings.

### Study design and patient samples

**Figure 1:**
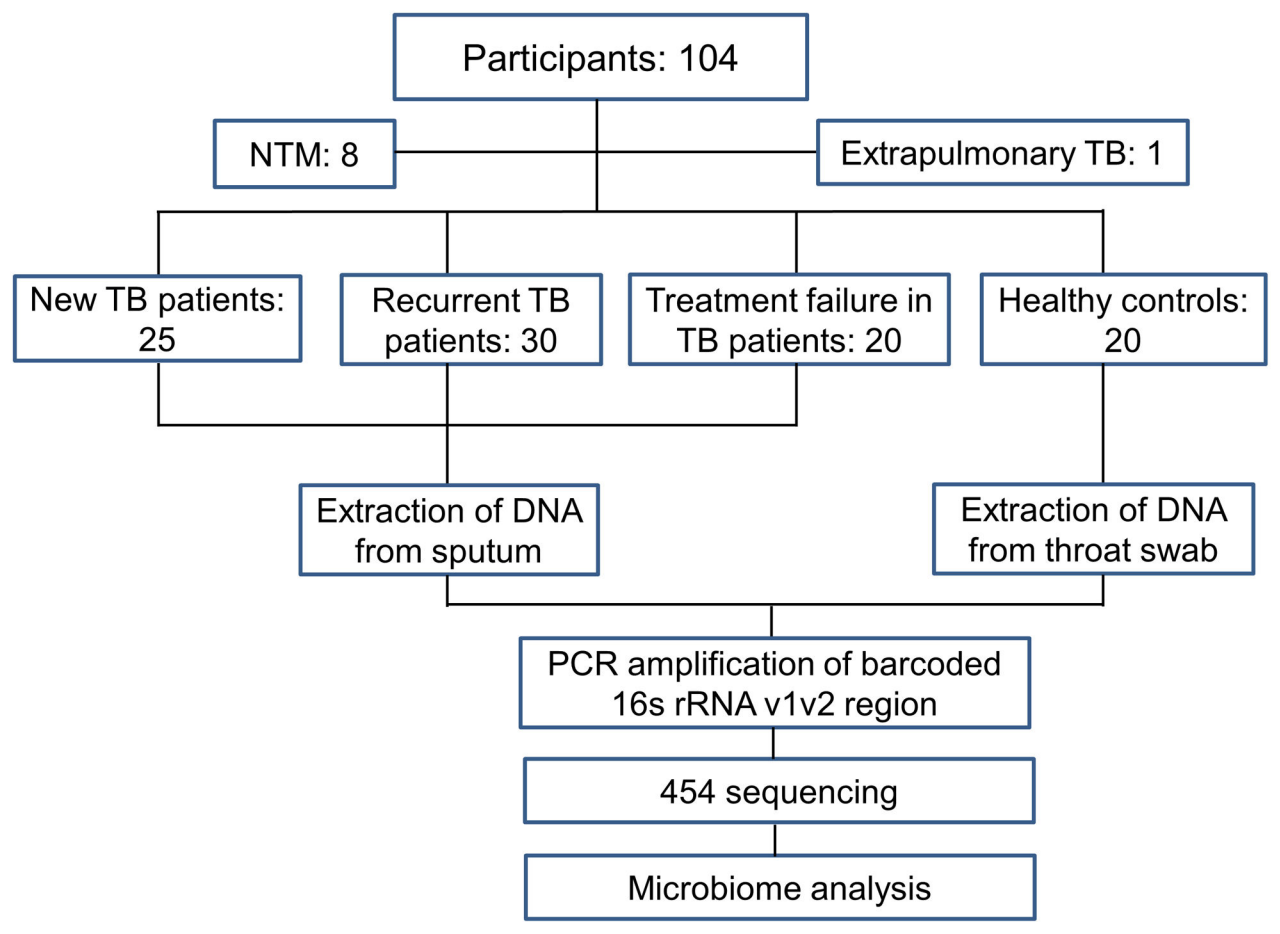
Flowchart of the study design. A total of 104 participants were recruited, and 95 were eligible for inclusion. Pulmonary TB patients were divided into three groups: New TB patients (N=25), recurrent TB patients (N=30), and treatment failure in TB patients (N=20). Participants without TB or other diseases served as healthy controls (N=20).

The current study analyzed the microbiota in different categories of pulmonary TB patients (Figure 1). A total of 84 TB patients and 20 healthy controls were initially enrolled from May 2011 to March 2012. Eight cases were excluded in final analysis after a diagnosis of NTM infections, and one of the patients was excluded due to a diagnosis of extrapulmonary TB. We divided the patients into three groups according to the following criteria. New TB group (N-TB, N=25): patients with newly developed pulmonary TB, in which 20 were cured after 6 month anti-TB treatment (cured new TB patients, C-TB, N=20). Recurrent TB group (N=30): Pulmonary TB patients who had previously been treated and declared cured prior to becoming once again bacteriologically positive. Treatment failure group (N=20): Smear-positive patients who remained smear positive at 5 months or more after initiation of treatment. Ethics approval was obtained by local IRB of Huashan Hospital, Fudan University.

Drug susceptibility testing for *M. tuberculosis* strains was performed for isoniazid (INH), rifampin (RIF), ethambutol (EMB) and streptomycin (SM) in Lowenstein-Jensen medium as described [14].

Sputum samples were collected from all the TB patients, and throat swab samples were obtained from 20 healthy volunteers before breakfast. DNA from sputum and throat swab samples was extracted using QIAGEN QIAamp DNA mini kit (QIAGEN, Valencia, CA). Briefly, DNA was isolated and purified per manufacturer’s recommendations through a spin column. The DNA was first adsorbed to the silica of the column followed by several washes with wash buffer containing 70% ethanol. Finally, the DNA was eluted and used for PCR amplification of 16S rRNA for microbiota identification by 454 sequencing.

### PCR amplification and 454 sequencing

The PCR enrichment of the 16S rRNA V1 and V2 hypervariable regions was performed using 8F (5’-AGAGTTTGATCCTGGCTCAG-3’) and 338R (5’-RTGCTGCCTCCCGTAGGAGT-3’) primer set [15,16]. The forward primers consisted of a directional GS FLX Titanium Primer A sequence and a 10 base pair barcode sequence upstream of the template specific 8F sequence to allow for sample identification, and the reverse primers consisted of a GS FLX Titanium Primer B sequence and 338R sequence. The barcoded primers used were listed in Table S1 in File S1. PCR cycling conditions were 94 °C for 5 min, followed by 30 cycles with denaturation at 94 °C for 30 s, anneal at 63 °C for 30 s and extension at 72 °C for 40 s using Ex Taq (Takara, Dalian) . The resulting 16S rRNA fragments were purified using MiniElute gel extraction kit (Qiagen). The DNA concentration in the purified PCR products was measured by PicoGreen quantification assay (Invitrogen). Barcoded samples were pooled with the same amount of DNA from each sample. The pooled PCR products were subjected to standard 454 DNA sequencing using routine protocols according to the GS FLX system manuals. The sequencing data were submitted to the NCBI Short Read Archive, and the accession number of the related BioProject is PRJNA215696.

### Data analysis and statistics

The 454 -generated FASTA file (.fna) and quality score file (.qual) were acquired as raw sequence data. The multiplexed reads were split and assigned to samples based on their unique nucleotide barcode. We screened, trimmed, and filtered the sequences using default settings of QIIME virtual machine version 1.5.0 (http://www.qiime.org) [17]. Qualified sequence fragments were assigned to taxonomic identities using reference databases from greengenes (http://greengenes.lbl.gov). The differences in taxonomic composition of microbial communities were analyzed by using Kruskal-Wallis test among three or four groups or Mann-Whitney U test between two groups (IBM SPSS Statistics, v20, and GraphPad Prism software Version 6.01). Alpha diversity and beta diversity measures were calculated and plotted using QIIME. Odds ratio (OR) was calculated and Fisher’s exact test was used to determine if the frequency distribution of the presence of a specific microorganism was associated with the TB disease state in STATA (StataCorp LP, version 9.0).

## Results

### Participant characteristics

Our primary objectives were to identify the lung microbiota that are associated with new TB patients and determine whether TB patients who had relapse or treatment failure have a microbiota profile that differ from new TB patients or healthy controls. The study design is shown in Figure 1. Ninety-five participants, including 25 new TB, 30 recurrent TB, 20 treatment failure patients, and 20 healthy people were recruited. Characteristics of enrolled participants are outlined in Table 1. Age and gender were similar among the TB patients but differed from healthy controls, which were younger than the TB patients (Mann-Whitney test, p<0.01). There was a higher percentage of male individuals in TB patient group (80.3%), especially in recurrent TB (86.7%) and treatment failure TB (90%). All patients included for further analysis were pulmonary TB. Moreover, the sputum culture-positive rate was lower in recurrent TB than either new TB or treatment failure patients. Monoresistance to INH or RIF was comparable and low in TB patients. MDR rates in recurrent TB and treatment failure were higher than that of new TB (26.7%&25% versus 7.6%), but the difference did not reach statistical significance. Among 25 new pulmonary TB patients, 20 cases were cured after 6 month DOTS therapy in follow-up. 

**Table 1:** Characteristics of the enrolled study participants.

	New tuberculosis (n=25)	Recurrent tuberculosis (n=30)	Treatment failure tuberculosis (n=20)	Healthy controls (n=20)
Mean age, years (range)	44 (13-77)	52 (22-79)	49 (20-78)	31 (24-55)
Sex (%)				
Male	16 (64%)	26 (86.7%)	18 (90%)	6 (30.0)
Female	9 (36%)	4 (13.3%)	2 (10%)	14 (70.0)
Site of TB				
Pulmonary TB	25 (100%)	30 (100%)	20 (100%)	
Smear positive (%)	25 (100%)	26 (86.7%)	20 (100%)	
Sputum-culture positive (%)	22 (88%)	12 (40%)	20 (100%)	
Drug susceptibility pattern (%)				
Monoresistance to Isoniazid	1 (4.0%)	0 (0.0)	2 (10%)	
Monoresistance to Rifampin	1 (4.0%)	5 (16.7%)	2 (10%)	
MDR	2 (8.0%)	8 (26.7%)	5 (25%)	

### Microbial diversity associated with healthy or TB disease state

**Figure 2:**
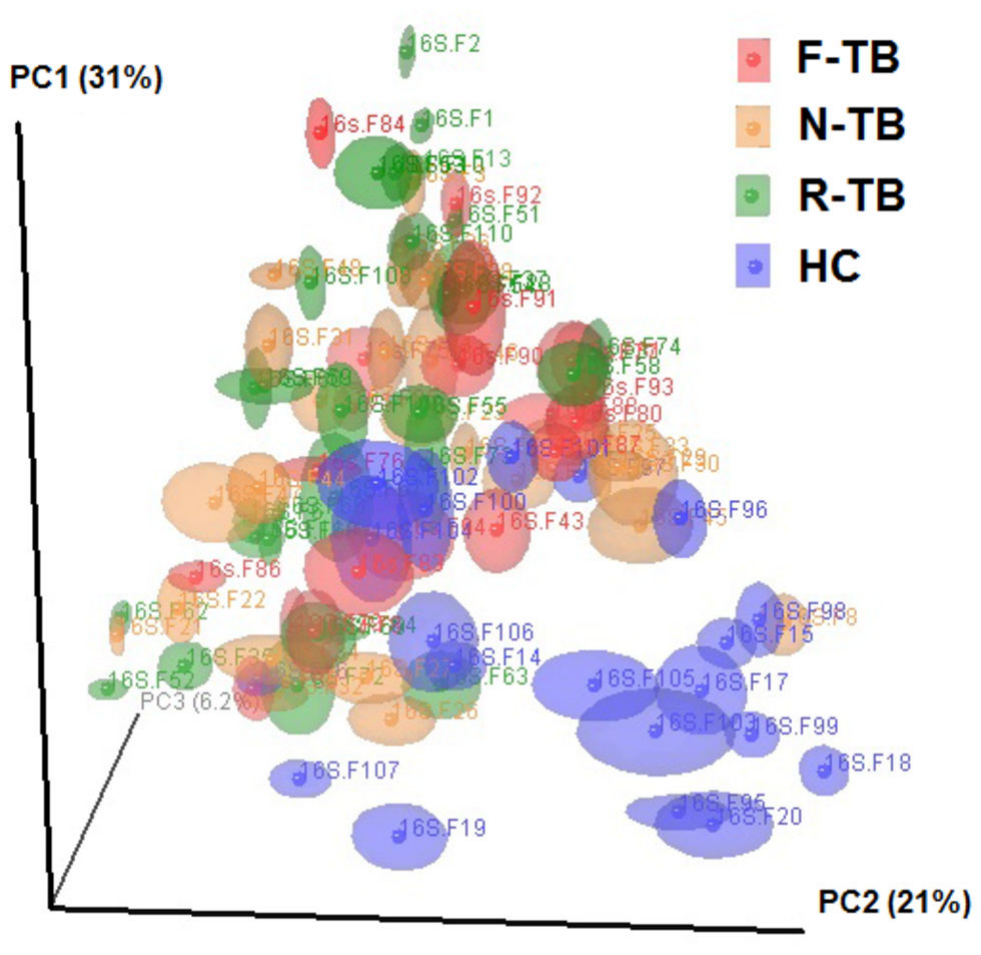
Principal Coordinates Analysis (PCoA) plot based on weighted UniFrac distance. Samples of N-TB (orange), R-TB (Green), and F-TB (Red) are shown as dots in the plot. F-TB, treatment failure TB; N-TB, new TB; R-TB, recurrent TB; HC, healthy controls.

To identify microbial diversity between a group of samples among communities (beta diversity), the microbial profiles were subjected to Principal Coordinates Analysis (PCoA) to determine if variations in microbiota could explain differences in outcomes of TB treatment. PcoAs of beta diversity among samples in different groups was generated from the phylogeny-based weighted (Figure 2) Unifrac distance metric. The majority of samples from healthy controls displayed differential clustering from that of TB samples, however, no obvious clustering was observed among three TB groups with different outcomes of TB treatment. To compute the within community diversity (alpha diversity), the rarefaction curves (Figure S1 in File S1) were generated based on phylogenetic metrics (Phylogenetic Diversity, PD whole tree). The microbiota in healthy controls represented the richest species diversity whereas the microbiota in recurrent TB represented the poorest species diversity. There was no significant difference among microbial communities when we performed Mann-Whitney U test to compare differences between either two groups based on samples rarefied to any fixed sequences per sample (data not shown).

### Microbial community in patient sputum samples

To analyze the microbiota associated with TB and different categories of TB compared with the healthy controls, sputum samples from the above subjects were subjected to 454 deep sequencing of the 16S rRNA gene as described in the Methods. The average raw sequence length passing quality filters was 377 bp, and the min/max/mean sample sequence count was 581/3106/1723. The reads were assigned to different taxonomic units by using the Ribosomal Database Project (RDP) classifier [18]. After demultiplexing, a total of 162,452 sequences included were classified into kingdom of bacteria, among which about 93.5% could be classified to genus level. The taxonomic summary information for each community at the phylum level and the genus level are shown in Figure 2. Each column summarizes sequences obtained from a separate DNA sample, grouped by sample ID. The relative abundance (in percentage) of each bacterial phylum and genus are shown by the color code. 

**Figure 3:**
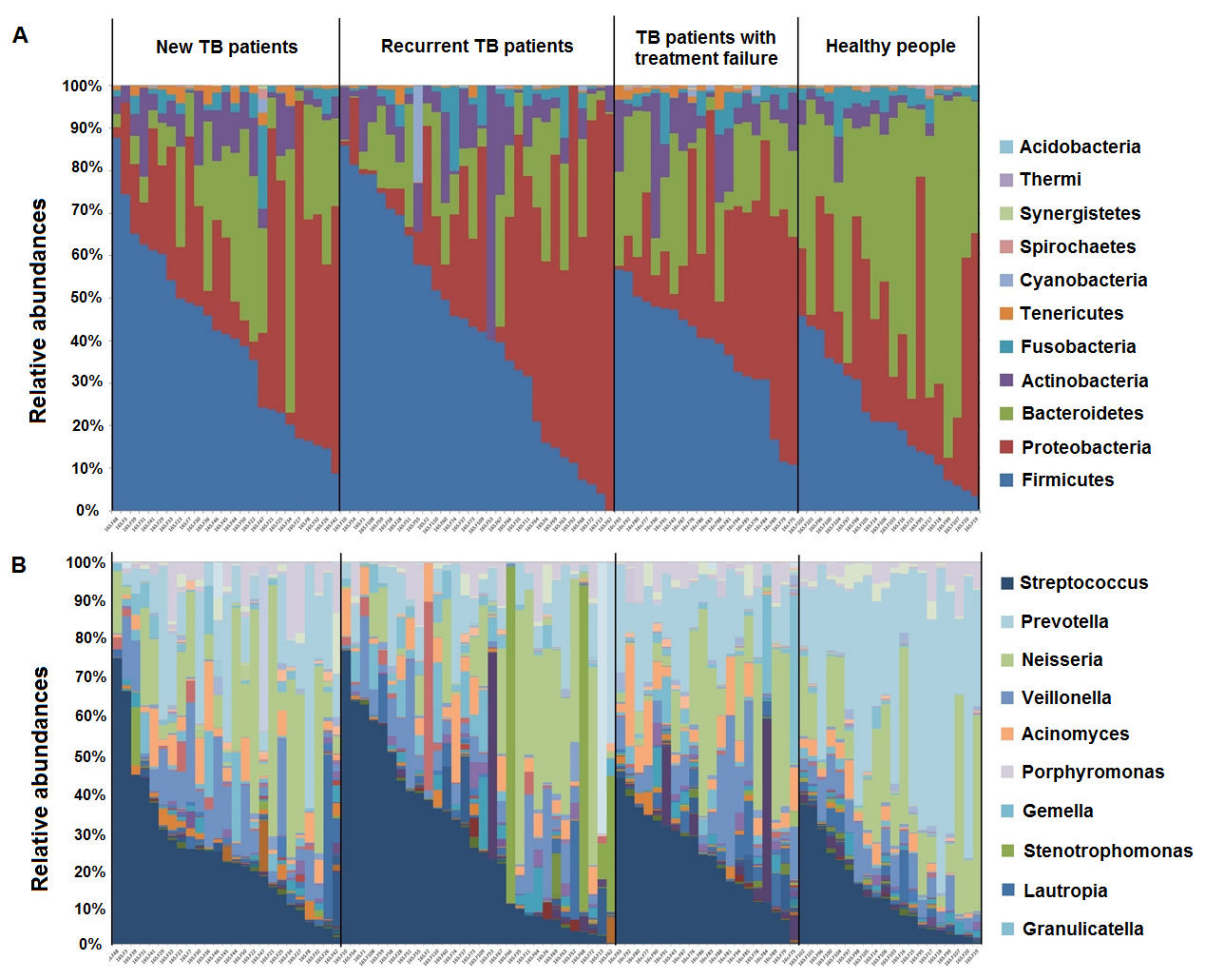
Relative abundance of microbial composition in different categoeis of TB patients and healthy controls. (a) Phylum level abundance profiles in each sample. Color band is sorted by the most abundant phylum *Firmicutes* in each group. All 11 detected phyla are listed as symbols. (b) Genus level abundance profiles in each sample. Color band was sorted by the most abundant genus *Streptococcus* in each group. Only the top 10 genera are shown in the figure, see Information S1in File S1 for additional details.

**Figure 4:**
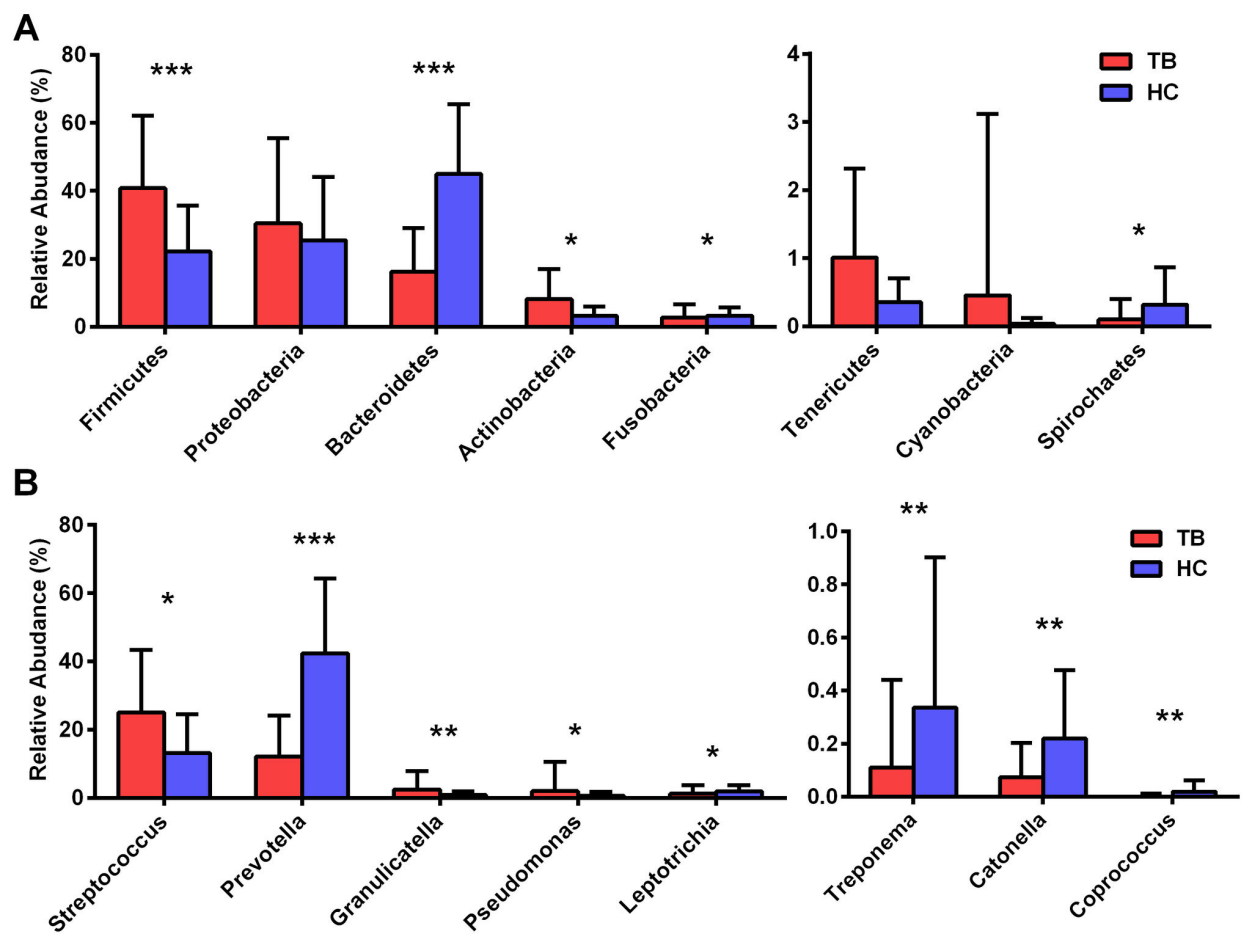
Mean relative abundance of microbial communities in tuberculosis (TB) patients and healthy controls (HC). (a) Relative abundance at phylum level with more than 0.5% in average in either TB or HC group. (b) Relative abundance of genera with statistical differences between TB patients and HC. *: p<0.05, **: p<0.01, ***: p<0.001. SD: standard deviation.

Sputum samples from TB patients and healthy controls had similar microbial community composition at the phylum level, but *Bacteroidetes* was more dominant in healthy controls than in TB patients (Figure 3). The differences in microbial abundance between TB patients and healthy controls are shown in Figure 4. At the phylum level, we detected 11 phyla in both TB patients and healthy controls, and statistically significant differences were observed in specific phyla such as *Firmicutes*, *Actinobacteria* and *Spirochaetes* as more abundant in TB than in healthy controls whereas *Bacteroidetes* and *Fusobacteria* were more abundant in healthy controls than in TB (Figure 4a). *Cyanobacteria*, *Thermi*, and *Acidobacteria* represented low abundance (less than 0.1% in average), and had no statistical significance in abundance between TB patients and healthy controls (data not shown). At the genus level, Figure 4b shows the genera with statistical differences in abundance between TB patients and healthy controls, where *Prevotella*, *Leptotrichia*, *Treponema*, *Catonella* and *Coprococcus* were more abundant in healthy controls than in TB patients. In contrast, genera such as *Streptococcus*, *Gramulicatella* and *Pseudomonas* were more abundant in TB patients than in healthy controls. Genera *Bergeyella* and *Sharpea* were identified to be unique to TB patients and were detected in more than 10% of the TB patients (Table S2 in File S1), and the genus *Haloplasma* was found more frequently in TB patients compared to healthy controls (p=0.063, Table S3 in File S1). 

### Abundance and existence of specific bacteria associated with TB disease state

**Figure 5:**
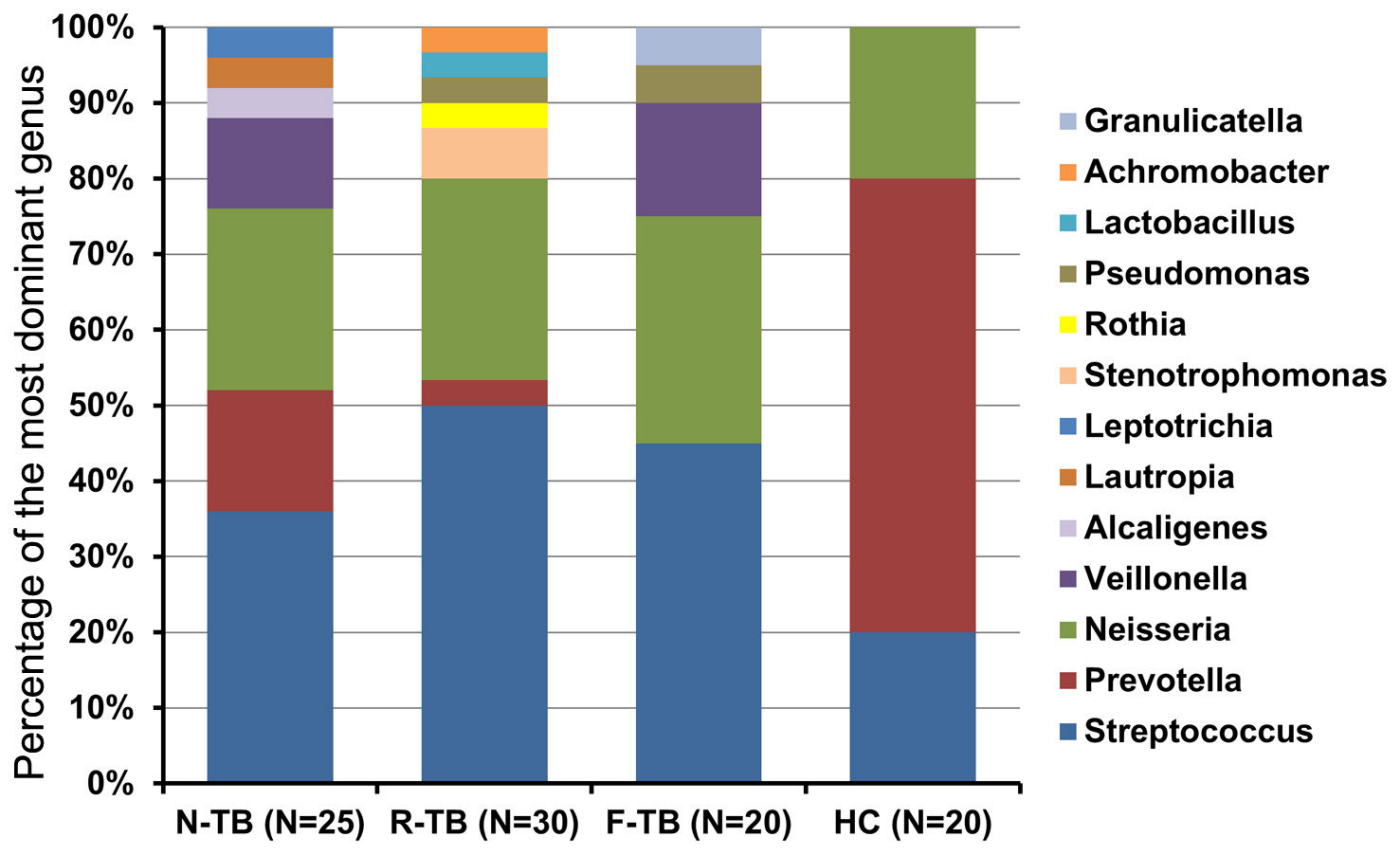
Distribution of the most dominant genus in different categories of TB patients versus healthy controls. Each column shows the relative percentage of the most dominant genus in each group. N-TB, new TB patients; R-TB, recurrent TB patients; F-TB treatment failure TB patients.

In the healthy control group, the majority of the cases were dominated by *Prevotella* (60%), followed by *Streptococcus* (20%) and *Neisseria* (20%). In the TB patient group, most of the cases were dominated by *Streptococcus* (36% in new TB, 50% in recurrent TB and 45% in treatment failure TB), followed by *Neisseria* (24% in new TB, 26.7% in recurrent TB and 30% in treatment failure TB). *Prevotella* was less common in TB patients (16% in new TB, 3.3% in recurrent TB and 0% in treatment failure TB) than in healthy controls. Some TB patients were more likely to be dominated by other genera in addition to *Prevotella*, *Streptococcus* and *Neisseria*, such as *Veillonella*, *Alcaligenes*, *Lautropia* and *Leptotrichia* in new TB, and *Achromobacter*, *Lactobacillus*, *Pseudomonas*, *Rothia* and *Stenotrophomonas* in recurrent TB, and *Granulicatella*, *Pseudomonas* and *Veillonella* in treatment failure patients (Figure 5, Table S4 in File S1).

**Figure 6:**
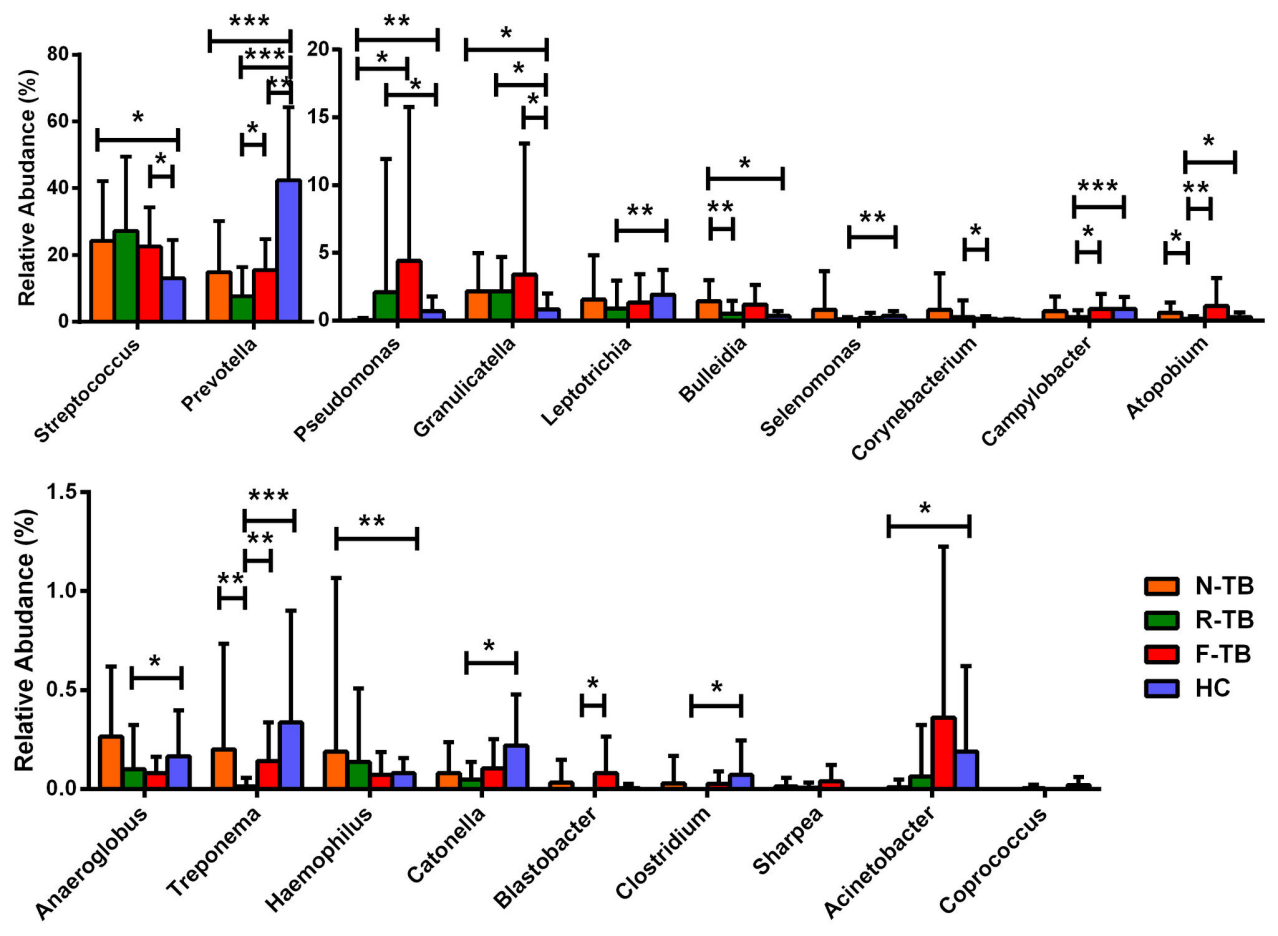
Mean relative abundance of genera with statistical difference among different groups. Mann–Whitney tests were performed between any two groups, and Kruskal–Wallis test were performed among four groups. N-TB, new TB patients; R-TB, recurrent TB patients; F-TB treatment failure TB patients; HC, healthy controls.

To determine if certain genera are associated with a particular category of the disease states, we analyzed the relative abundance of specific bacteria with significant differences among groups with various TB disease states. As shown in Figure 6, the abundance of *Streptococcus*, *Granulicatella*, *Bulleidia* and *Haemophilus* were higher in new TB than those in healthy controls, while the abundance of *Prevotella*, *Pseudomonas* and *Acinetobacter* were lower in new TB than those in healthy controls; the abundance of *Pseudomonas* was higher in recurrent TB than that in healthy controls, while the abundance of *Prevotella*, *Granulicatella*, *Leptotrichia*, *Selenomonas*, *Campylobacter*, *Atopobium*, *Anaeroglobus*, *Treponema*, *Catonella* and *Clostridium* were lower in recurrent TB than those in healthy controls; the abundance of *Streptococcus* and *Granulicatella* were higher in treatment failure TB than those in healthy controls. *Bulleidia*, *Atopobium* and *Treponema* were less abundant in recurrent TB than in new TB; *Pseudomonas* was more abundant in treatment failure TB than in new TB. *Corynebacterium* was more abundant in recurrent TB than in treatment failure TB, while *Prevotella*, *Campylobacter*, *Atopobium*, *Treponema* and *Blastobacter* were more abundant in treatment failure TB than in recurrent TB. We observed that the genera *Atopobium* and *Treponema* were lowest in recurrent TB patients among all the groups with statistical significance. *Sharpea* and *Coprococcus* showed no difference between any two groups (new TB, recurrent TB, treatment failure TB and healthy controls) using Mann-Whitney U test, but they differed among four groups when compared using Kruskal–Wallis test (p=0.045 and p=0.01, respectively). 

To examine if the existence of some specific microorganisms may be associated with TB disease states, we set 1% as a cutoff level for abundance detection for the 454 sequencing data. The cutoff 1% was chosen because the depth of coverage of about 1000 sequences / sample allows us to infer the frequency of populations at 1% abundance with reasonable accuracy [19]. The positive sample counts and odds ratio (OR) between new TB patients and recurrent TB patients, and between cured new TB patients (C-TB) and treatment failure TB patients are listed in Table 2 with a p value less than 0.05. *Bulleidia* and *Atopobium* were significantly less frequent in recurrent TB than in new TB (P=0.005 and p=0.002) (Table 2) and also less abundant in R-TB than in N-TB (Figure 6). *Pseudomonas* was more frequently present in treatment failure TB than in cured TB (p=0.008, Table 2), and this genus was also more abundant in treatment failure TB than in new TB (Figure 6). 

**Table 2:** OR and significance of existence of some specific microorganisms between recurrent TB patients and new TB patients, and between treatment failure patients and cured patients with positive cutoff of 1%.

**Genus**	**R-TB**	**N-TB**	**OR (95%CI)**	**Fisher's exact p value**
Recurrent TB patients versus new TB patients				
***Bulleidia***	3 (10%)	11 (44%)	0.14 (0.02-0.68)	0.005
***Atopobium***	0	7 (28%)	0 (0-0.36)	0.002
Treatment failure TB patients versus cured new TB patients				
***Pseudomonas***	7 (35%)	0	NA	0.008

Abbreviations: R-TB, recurrent TB patients; N-TB, new TB patients; F-TB, TB patients with treatment failure; C-TB, cured new TB patients; OR, odds ratio; CI, confidence interval; NA: not available.

**Figure 7:**
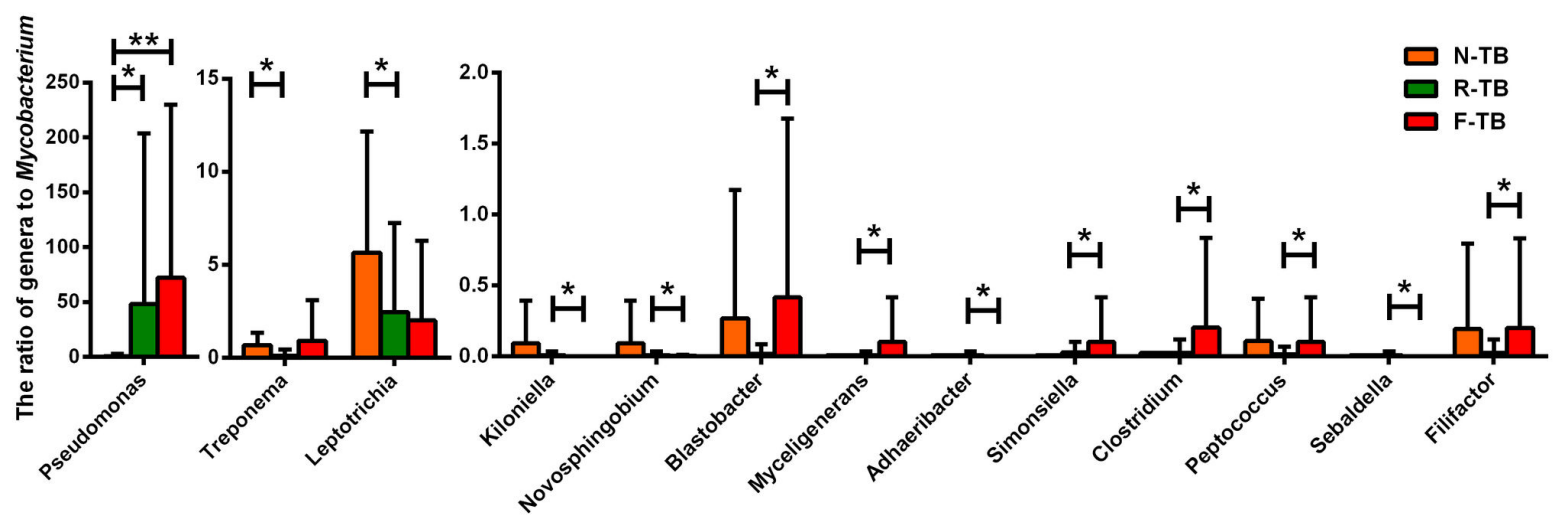
The ratio of genera to *Mycobacterium*. The ratio of each detected genus to *Mycobacterium* was calculated, and Mann–Whitney tests were performed between any two groups. Genera with statistical differences were listed. N-TB, new TB patients; R-TB, recurrent TB patients; F-TB treatment failure TB patients.

To examine if the abundance of the different genera may be associated with the abundance of *Mycobacterium* in different groups, we calculated the Genera / *Mycobacterium* ratio to see if the abundance of special genera would affect the existence of *Mycobacteria*. Only 48% (12 of 25) in new TB, 23.3% (7 of 30) in recurrent TB and 50% (10 of 20) in treatment failure TB could detect the existence of *Mycobacterium*, which were used for further analysis (Figure 7). The ratio of *Pseudomonas* / *Mycobacterium* in recurrent TB and treatment failure TB were higher than that in new TB (p<0.001), which is consistent with the abundance results in Figure 6. The ratio of *Treponema* / *Mycobacterium* in recurrent TB was lower than that in new TB (p=0.011), which is also consistent with the results in Figure 6. 

## Discussion

Although sputum culture is the most common method for definitive diagnosis of TB [20,21], this traditional culture-based diagnosis cannot estimate the microbial communities present during *Mtb* infection. New deep sequencing technologies now enable comprehensive examination of microbial communities, and sputum microbiota structure has been analyzed in a variety of studies from patients with asthma, cystic fibrosis and lower respiratory tract infections [9,11,22,23]. Two recently published studies reported the composition of sputum microbiota in TB patients, but these studies used a relatively small number of patient samples and only looked at sputum microbiota from patients with active disease. The present study has characterized microbiota in the sputa of TB patients associated with *Mtb* infection and also the microorganisms associated with the different outcomes of treatment. Our study sampled a relatively large cohort of participants (n=95) compared to earlier studies (22 TB patients in [12] and 31 TB patients in [13]), and the most important contribution of this work is the first comprehensive analysis of sputum microbiota in TB patients with various disease states.

In this study, we analyzed the differences of genera in abundance, frequency, as well as the association of abundance to *Mycobacterium* among different TB diseased groups. We found that *Pseudomonas* is the only genus that is more abundant or more frequently present in TB treatment failure patients or recurrent TB than in new or cured TB patients and healthy controls (Figure 6, Table 2), and it is also the only genus differentially existed between cured TB patients and treatment failure patients. Furthermore, the ratio of *Pseudomonas* / *Mycobacterium* in treatment failure was higher than that in new TB (Figure 7). *Pseudomonas aeruginosa* is considered as the leading cause of morbidity and mortality in cystic fibrosis as well as chronic obstructive lung disease [24,25]. *Pseudomonas* sp. monoinfection is associated with a reduction of the microbial diversity in chronic obstructive pulmonary disease patients [26]. It seems to be very possible to associate with poor prognosis especially with treatment failure. However, which species of *Pseudomonas* are mainly associated with the TB recurrence or treatment failure remains to be determined in future study. Besides *Pseudomonas*, some other specific genera also showed differences among various TB disease states. *Prevotella*, a well identified genus of bacterial flora of oral cavity, was especially low in recurrent TB patients compared with healthy controls and other TB patients. We also found reduced abundance of *Bulleidia*, *Atopobium* and *Treponema* in recurrent TB patients compared with those in new TB patients (Figure 6). Moreover, *Bulleidia* and *Atopobium* were less frequently found in recurrent TB than in new TB patients (Table 2). These genera have been previously found in oral cavity in healthy subjects [27], and can be defined as normal flora in humans. The difference in abundance and frequency of these genera in recurrent TB and treatment failure patients may suggest that the disruption of normal bacterial flora in respiratory tract could be one of the risk factors of poor prognosis of TB treatment. There were also some genera with statistically different abundance between recurrent TB and treatment failure TB. *Corynebacterium*, which was more abundant in recurrent TB than treatment failure TB, could be found in respiratory tracts, and some of its species were the pathogens of diphtheria and other respiratory diseases [28,29]. *Prevotella*, *Campylobacter*, *Atopobium*, *Treponema* and *Blastobacter* were genera presenting less abundant in recurrent TB than in treatment failure TB. *Campylobacter* was reported to be pathogen in respiratory infections in tropics subtropics and related to travel-associated illness [30,31]. Mixed infections with some specific bacteria such as *Treponema* cause severe respiratory disease state, as demonstrated by a mouse pneumonia model [32]. There were no reports till now showing *Blastobacter* could induce any respiratory disease. The disruption of sputum bacteria may be a risk factor of poor prognosis of TB treatment, but further work is needed to clarify if mixed infection or absence of some bacteria is underlying complex disease state.

We have found that sputum microbiome of TB patients was distinct from that of the healthy controls from the Unifrac distance analysis, which was similar to the study by Cui et al. [12]. However, the TB sputum microbiome in Cui’s study has a higher diversity than that in healthy controls [12] whereas we found that healthy microbiome is more diverse than the TB groups though without statistical significance. Several factors may help to explain the discrepancy. One possibility is that we may have detected more minor bacterial species with a higher sequencing depth (1723) than that in Cui’s study (1307) [12]. In addition, one of the reasons could be under-sampling and future studies may increase the sample size for comparison. Another possibility is the sampling differences between the two studies, although the samples and controls in Cui’s study and ours were comparable. However, the study by Cheung et al. that recruited individuals with coughing symptoms as controls, claimed no significant differences in diversity between controls and TB sputum microbiomes [13]. It is possible that different choices of controls for comparison may have affected the bacterial species being identified from these studies. 

The existence of airway microbiota which included *Streptococcus*, *Prevotella*, *Veillonella* and *Neisseria* is consistent with other published findings [8,11,13]. However, we found differences in relative abundance of some special microbial composition between TB patients and healthy controls (Figure 4). Genera *Streptococcus*, *Granulicatella*, *Actinomyces*, *Prevotella*, and *Veillonella* were predominant in both TB patients and healthy controls, which is consistent with recent studies [12,13]. *Stenotrophomonas* and *Pseudomonas* were found to be unique in TB patients in a previous study [12], but in our study these genera were found in healthy controls with percentage of 35% and 55%, respectively. Genera *Moryella*, *Mogibacterium* and *Oribacterium* were found statistically enriched in TB patients in the study by Cheung et al. [13], but not in our study or the study by Cui et al. [12]. Moreover, all the samples from healthy controls and majority of samples from the TB patients were usually dominated by *Prevotella*, *Streptococcus* or *Neisseria*, but some other genera such as *Veillonella*, *Stenotrophomonas* and *Pseudomonas* were dominant in sputum samples of some TB patients (Figure 5).

The successful anti-TB therapy can not kill all *M. tuberculosis* bacteria in many cured TB patients, and the disease can go to a latent stage where host immune responses help to control the growth of the mycobacteria. Recent studies reveal that innate immune recognition by pattern-recognition receptors of the microbiota promotes host-microbial symbiosis [33]. Probiotics in the gut and other organs have the ability to activate dendritic cells and monocytes/macrophages by interacting with Toll-like receptors [34,35], and dendritic cell expression of the signaling molecule TRAF6 seems to be critical for gut microbiota-dependent immune tolerance [36]. Disorder in respiratory microbiota, including the invasion of foreign pathogens, or reduction of probiotics may result in the disruption of immune balance leading to disease activation or becoming more susceptible to new infections [26,32] or may interfere with cure of the disease. 

There are some limitations in our study. First, the relatively low sequencing depth with an average of 1723 reads per sample, may miss some less abundant bacterial genera compared to other studies. However, it has been demonstrated that depth of coverage of 1000 sequences / sample (lower than ours) to infer the frequency of bacterial populations with reasonable accuracy [19]. Second, the sampling method using throat swabs for healthy controls compared with the sampling sputum in TB patients may make it difficult to determine if the observed differences between the two groups represent the real difference. However, this is a problem that is not easily addressed as it is difficult to obtain sputum specimens from healthy controls who usually do not produce sputum while getting bronchial lavage is hardly possible for healthy people. This potential problem is not unique to this study as the other study by Cui et al. [12] also used the same approach, and the study by Cheung et al. [13] that used sputum from “TB-resembling coughing symptoms” as controls is not ideal either and may also introduce bias. Nevertheless, recent studies revealed that microbial communities from upper respiratory tract and lower respiratory tract are closely related in healthy subjects and in patients with lung diseases [7,37], and these findings support that bacterial populations in healthy upper respiratory tract may largely reflect lower respiratory tract organisms. Another limitation is the sequencing length in our study (~330bp) was not long enough to map the majority of sequences to a species level. Next generation sequencing technology with a longer sequence read may help to overcome this problem in future studies. Since the 16S rRNA sequence data were generated from very short variable region, genera specific to disease conditions need to be checked by traditional PCR sequencing, or by real-time PCR (RT-PCR) using primers for genus specific loci, which will not only confirm that genus but also validate the copy number from deep sequencing data.

In conclusion, this study characterized the microbiota in pulmonary TB patients of different treatment outcomes and identified microbial genera that are associated with different categories of TB disease and may influence disease progression and treatment outcome. The presence of some specific bacteria may be associated with treatment outcome, such as *Pseudomonas* which is more likely to be present and abundant in recurrent TB patients and treatment failure TB patients than in new patients. Furthermore, our study also found the reduced presence or abundance of some bacteria in recurrent TB, such as *Treponema* and *Atopobium*, which indicate that disorder of normal microbiome may be a risk factor of TB recurrence. Future studies are needed to evaluate these findings in more patients and to assess the role of the identified bacterial genera in disease onset and pathogenesis in animal studies and test the possibility of treating these microbiota bacteria associated with TB, recurrent TB and treatment failure for improved treatment. 

## Supporting Information

File S1 Figure S1, Rarefaction analysis of 16S rRNA gene sequences of 3 groups of TB patients compared with healthy controls. F-TB, TB patients with treatment failure; HC, healthy controls; N-TB, new TB patients; R-TB, recurrent TB patients. Table S1, List of barcoded primers used in the study. Table S2, The existence of genera which were uniquely found in the sputum of TB patients. Table S3, OR and significance of existence of some specific microorganisms between TB patients and healthy controls. Table S4, Distribution of the most abundant genus in each group. Information S1, detailed figure legend in Figure 3b.
(DOC)
