# Supplementary material for: Sputum Microbiota Associated with New, Recurrent and Treatment Failure Tuberculosis
Source: PLoS One. 2013 Dec 13;8(12):e83445. doi: 10.1371/journal.pone.0083445 (PMC3862690; doi:10.1371/journal.pone.0083445)

# Supporting Information

**Table S1: List of barcoded primers used in the study**

| **Primer Name** | **Primer Sequences** |
| --- | --- |
| **16S-F1** | CGTATCGCCTCCCTCGCGCCATCAGACGAGTGCGTagagtttgatcctggctcag |
| **16S-F2** | CGTATCGCCTCCCTCGCGCCATCAGACGCTCGACAagagtttgatcctggctcag |
| **16S-F3** | CGTATCGCCTCCCTCGCGCCATCAGAGACGCACTCagagtttgatcctggctcag |
| **16S-F4** | CGTATCGCCTCCCTCGCGCCATCAGAGCACTGTAGagagtttgatcctggctcag |
| **16S-F5** | CGTATCGCCTCCCTCGCGCCATCAGATCAGACACGagagtttgatcctggctcag |
| **16S-F6** | CGTATCGCCTCCCTCGCGCCATCAGATATCGCGAGagagtttgatcctggctcag |
| **16S-F7** | CGTATCGCCTCCCTCGCGCCATCAGCGTGTCTCTAagagtttgatcctggctcag |
| **16S-F8** | CGTATCGCCTCCCTCGCGCCATCAGCTCGCGTGTCagagtttgatcctggctcag |
| **16S-F10** | CGTATCGCCTCCCTCGCGCCATCAGTCTCTATGCGagagtttgatcctggctcag |
| **16S-F11** | CGTATCGCCTCCCTCGCGCCATCAGTGATACGTCTagagtttgatcctggctcag |
| **16S-F13** | CGTATCGCCTCCCTCGCGCCATCAGCATAGTAGTGagagtttgatcctggctcag |
| **16S-F14** | CGTATCGCCTCCCTCGCGCCATCAGCGAGAGATACagagtttgatcctggctcag |
| **16S-F15** | CGTATCGCCTCCCTCGCGCCATCAGATACGACGTAagagtttgatcctggctcag |
| **16S-F16** | CGTATCGCCTCCCTCGCGCCATCAGTCACGTACTAagagtttgatcctggctcag |
| **16S-F17** | CGTATCGCCTCCCTCGCGCCATCAGCGTCTAGTACagagtttgatcctggctcag |
| **16S-F18** | CGTATCGCCTCCCTCGCGCCATCAGTCTACGTAGCagagtttgatcctggctcag |
| **16S-F19** | CGTATCGCCTCCCTCGCGCCATCAGTGTACTACTCagagtttgatcctggctcag |
| **16S-F20** | CGTATCGCCTCCCTCGCGCCATCAGACGACTACAGagagtttgatcctggctcag |
| **16S-F21** | CGTATCGCCTCCCTCGCGCCATCAGCGTAGACTAGagagtttgatcctggctcag |
| **16S-F22** | CGTATCGCCTCCCTCGCGCCATCAGTACGAGTATGagagtttgatcctggctcag |
| **16S-F23** | CGTATCGCCTCCCTCGCGCCATCAGTACTCTCGTGagagtttgatcctggctcag |
| **16S-F24** | CGTATCGCCTCCCTCGCGCCATCAGTAGAGACGAGagagtttgatcctggctcag |
| **16S-F25** | CGTATCGCCTCCCTCGCGCCATCAGTCGTCGCTCGagagtttgatcctggctcag |
| **16S-F26** | CGTATCGCCTCCCTCGCGCCATCAGACATACGCGTagagtttgatcctggctcag |
| **16S-F27** | CGTATCGCCTCCCTCGCGCCATCAGACGCGAGTATagagtttgatcctggctcag |
| **16S-F28** | CGTATCGCCTCCCTCGCGCCATCAGACTACTATGTagagtttgatcctggctcag |
| **16S-F29** | CGTATCGCCTCCCTCGCGCCATCAGACTGTACAGTagagtttgatcctggctcag |
| **16S-F30** | CGTATCGCCTCCCTCGCGCCATCAGAGACTATACTagagtttgatcctggctcag |
| **16S-F31** | CGTATCGCCTCCCTCGCGCCATCAGAGCGTCGTCTagagtttgatcctggctcag |
| **16S-F32** | CGTATCGCCTCCCTCGCGCCATCAGAGTACGCTATagagtttgatcctggctcag |
| **16S-F33** | CGTATCGCCTCCCTCGCGCCATCAGATAGAGTACTagagtttgatcctggctcag |
| **16S-F34** | CGTATCGCCTCCCTCGCGCCATCAGCACGCTACGTagagtttgatcctggctcag |
| **16S-F35** | CGTATCGCCTCCCTCGCGCCATCAGCAGTAGACGTagagtttgatcctggctcag |
| **16S-F36** | CGTATCGCCTCCCTCGCGCCATCAGCGACGTGACTagagtttgatcctggctcag |
| **16S-F37** | CGTATCGCCTCCCTCGCGCCATCAGTACACACACTagagtttgatcctggctcag |
| **16S-F38** | CGTATCGCCTCCCTCGCGCCATCAGTACACGTGATagagtttgatcctggctcag |
| **16S-F39** | CGTATCGCCTCCCTCGCGCCATCAGTACAGATCGTagagtttgatcctggctcag |
| **16S-F40** | CGTATCGCCTCCCTCGCGCCATCAGTACGCTGTCTagagtttgatcctggctcag |
| **16S-F41** | CGTATCGCCTCCCTCGCGCCATCAGTAGTGTAGATagagtttgatcctggctcag |
| **16S-F42** | CGTATCGCCTCCCTCGCGCCATCAGTCGATCACGTagagtttgatcctggctcag |
| **16S-F43** | CGTATCGCCTCCCTCGCGCCATCAGTCGCACTAGTagagtttgatcctggctcag |
| **16S-F44** | CGTATCGCCTCCCTCGCGCCATCAGTCTAGCGACTagagtttgatcctggctcag |
| **16S-F45** | CGTATCGCCTCCCTCGCGCCATCAGTCTATACTATagagtttgatcctggctcag |
| **16S-F46** | CGTATCGCCTCCCTCGCGCCATCAGTGACGTATGTagagtttgatcctggctcag |
| **16S-F47** | CGTATCGCCTCCCTCGCGCCATCAGTGTGAGTAGTagagtttgatcctggctcag |
| **16S-F48** | CGTATCGCCTCCCTCGCGCCATCAGACAGTATATAagagtttgatcctggctcag |
| **16S-F49** | CGTATCGCCTCCCTCGCGCCATCAGACGCGATCGAagagtttgatcctggctcag |
| **16S-F50** | CGTATCGCCTCCCTCGCGCCATCAGACTAGCAGTAagagtttgatcctggctcag |
| **16S-F51** | CGTATCGCCTCCCTCGCGCCATCAGAGCTCACGTAagagtttgatcctggctcag |
| **16S-F52** | CGTATCGCCTCCCTCGCGCCATCAGAGTATACATAagagtttgatcctggctcag |
| **16S-F53** | CGTATCGCCTCCCTCGCGCCATCAGAGTCGAGAGAagagtttgatcctggctcag |
| **16S-F54** | CGTATCGCCTCCCTCGCGCCATCAGAGTGCTACGAagagtttgatcctggctcag |
| **16S-F55** | CGTATCGCCTCCCTCGCGCCATCAGCGATCGTATAagagtttgatcctggctcag |
| **16S-F56** | CGTATCGCCTCCCTCGCGCCATCAGCGCAGTACGAagagtttgatcctggctcag |
| **16S-F57** | CGTATCGCCTCCCTCGCGCCATCAGCGCGTATACAagagtttgatcctggctcag |
| **16S-F58** | CGTATCGCCTCCCTCGCGCCATCAGCGTACAGTCAagagtttgatcctggctcag |
| **16S-F59** | CGTATCGCCTCCCTCGCGCCATCAGCGTACTCAGAagagtttgatcctggctcag |
| **16S-F60** | CGTATCGCCTCCCTCGCGCCATCAGCTACGCTCTAagagtttgatcctggctcag |
| **16S-F61** | CGTATCGCCTCCCTCGCGCCATCAGCTATAGCGTAagagtttgatcctggctcag |
| **16S-F62** | CGTATCGCCTCCCTCGCGCCATCAGTACGTCATCAagagtttgatcctggctcag |
| **16S-F63** | CGTATCGCCTCCCTCGCGCCATCAGTAGTCGCATAagagtttgatcctggctcag |
| **16S-F64** | CGTATCGCCTCCCTCGCGCCATCAGTATATATACAagagtttgatcctggctcag |
| **16S-F65** | CGTATCGCCTCCCTCGCGCCATCAGTATGCTAGTAagagtttgatcctggctcag |
| **16S-F66** | CGTATCGCCTCCCTCGCGCCATCAGTCACGCGAGAagagtttgatcctggctcag |
| **16S-F67** | CGTATCGCCTCCCTCGCGCCATCAGTCGATAGTGAagagtttgatcctggctcag |
| **16S-F68** | CGTATCGCCTCCCTCGCGCCATCAGTCGCTGCGTAagagtttgatcctggctcag |
| **16S-F69** | CGTATCGCCTCCCTCGCGCCATCAGTCTGACGTCAagagtttgatcctggctcag |
| **16S-F70** | CGTATCGCCTCCCTCGCGCCATCAGTGAGTCAGTAagagtttgatcctggctcag |
| **16S-F71** | CGTATCGCCTCCCTCGCGCCATCAGTGTAGTGTGAagagtttgatcctggctcag |
| **16S-F72** | CGTATCGCCTCCCTCGCGCCATCAGTGTCACACGAagagtttgatcctggctcag |
| **16S-F73** | CGTATCGCCTCCCTCGCGCCATCAGTGTCGTCGCAagagtttgatcctggctcag |
| **16S-F74** | CGTATCGCCTCCCTCGCGCCATCAGACACATACGCagagtttgatcctggctcag |
| **16S-F75** | CGTATCGCCTCCCTCGCGCCATCAGACAGTCGTGCagagtttgatcctggctcag |
| **16S-F76** | CGTATCGCCTCCCTCGCGCCATCAGACATGACGACagagtttgatcctggctcag |
| **16S-F77** | CGTATCGCCTCCCTCGCGCCATCAGACGACAGCTCagagtttgatcctggctcag |
| **16S-F78** | CGTATCGCCTCCCTCGCGCCATCAGACGTCTCATCagagtttgatcctggctcag |
| **16S-F79** | CGTATCGCCTCCCTCGCGCCATCAGACTCATCTACagagtttgatcctggctcag |
| **16S-F80** | CGTATCGCCTCCCTCGCGCCATCAGACTCGCGCACagagtttgatcctggctcag |
| **16S-F81** | CGTATCGCCTCCCTCGCGCCATCAGAGAGCGTCACagagtttgatcctggctcag |
| **16S-F82** | CGTATCGCCTCCCTCGCGCCATCAGAGCGACTAGCagagtttgatcctggctcag |
| **16S-F83** | CGTATCGCCTCCCTCGCGCCATCAGAGTAGTGATCagagtttgatcctggctcag |
| **16S-F84** | CGTATCGCCTCCCTCGCGCCATCAGAGTGACACACagagtttgatcctggctcag |
| **16S-F85** | CGTATCGCCTCCCTCGCGCCATCAGAGTGTATGTCagagtttgatcctggctcag |
| **16S-F86** | CGTATCGCCTCCCTCGCGCCATCAGATAGATAGACagagtttgatcctggctcag |
| **16S-F87** | CGTATCGCCTCCCTCGCGCCATCAGATATAGTCGCagagtttgatcctggctcag |
| **16S-F88** | CGTATCGCCTCCCTCGCGCCATCAGATCTACTGACagagtttgatcctggctcag |
| **16S-F89** | CGTATCGCCTCCCTCGCGCCATCAGCACGTAGATCagagtttgatcctggctcag |
| **16S-F90** | CGTATCGCCTCCCTCGCGCCATCAGCACGTGTCGCagagtttgatcctggctcag |
| **16S-F91** | CGTATCGCCTCCCTCGCGCCATCAGCATACTCTACagagtttgatcctggctcag |
| **16S-F92** | CGTATCGCCTCCCTCGCGCCATCAGCGACACTATCagagtttgatcctggctcag |
| **16S-F93** | CGTATCGCCTCCCTCGCGCCATCAGCGAGACGCGCagagtttgatcctggctcag |
| **16S-F94** | CGTATCGCCTCCCTCGCGCCATCAGCGTATGCGACagagtttgatcctggctcag |
| **16S-F95** | CGTATCGCCTCCCTCGCGCCATCAGCGTCGATCTCagagtttgatcctggctcag |
| **16S-F96** | CGTATCGCCTCCCTCGCGCCATCAGCTACGACTGCagagtttgatcctggctcag |
| **16S-F97** | CGTATCGCCTCCCTCGCGCCATCAGCTAGTCACTCagagtttgatcctggctcag |
| **16S-F98** | CGTATCGCCTCCCTCGCGCCATCAGCTCTACGCTCagagtttgatcctggctcag |
| **16S-F99** | CGTATCGCCTCCCTCGCGCCATCAGCTGTACATACagagtttgatcctggctcag |
| **16S-F100** | CGTATCGCCTCCCTCGCGCCATCAGTAGACTGCACagagtttgatcctggctcag |
| **16S-F101** | CGTATCGCCTCCCTCGCGCCATCAGTAGCGCGCGCagagtttgatcctggctcag |
| **16S-F102** | CGTATCGCCTCCCTCGCGCCATCAGTAGCTCTATCagagtttgatcctggctcag |
| **16S-F103** | CGTATCGCCTCCCTCGCGCCATCAGTATAGACATCagagtttgatcctggctcag |
| **16S-F104** | CGTATCGCCTCCCTCGCGCCATCAGTATGATACGCagagtttgatcctggctcag |
| **16S-F105** | CGTATCGCCTCCCTCGCGCCATCAGTCACTCATACagagtttgatcctggctcag |
| **16S-F106** | CGTATCGCCTCCCTCGCGCCATCAGTCATCGAGTCagagtttgatcctggctcag |
| **16S-F107** | CGTATCGCCTCCCTCGCGCCATCAGTCGAGCTCTCagagtttgatcctggctcag |
| **16S-F108** | CGTATCGCCTCCCTCGCGCCATCAGTCGCAGACACagagtttgatcctggctcag |
| **16S-F109** | CGTATCGCCTCCCTCGCGCCATCAGTCTGTCTCGCagagtttgatcctggctcag |
| **16S-F110** | CGTATCGCCTCCCTCGCGCCATCAGTGAGTGACGCagagtttgatcctggctcag |
| **16S-R** | CTATGCGCCTTGCCAGCCCGCTCAGtgctgcctcccgtaggagt |

**Table S**2. The existence of genera which were uniquely found in the sputum of TB patients

| **Genara** | **N** | **α** | **β** |
| --- | --- | --- | --- |
| Bergeyella | 10 | 13.3% | 2.0% |
| Sharpea | 9 | 12.0% | 1.7% |
| Methyloversatilis | 5 | 6.7% | 0.8% |
| Weissella | 5 | 6.7% | 0.9% |
| Kiloniella | 4 | 5.3% | 0.7% |
| Novosphingobium | 4 | 5.3% | 0.7% |
| Johnsonella | 4 | 5.3% | 0.7% |
| Actinobacillus | 4 | 5.3% | 1.2% |
| Asteroleplasma | 4 | 5.3% | 2.5% |
| Enterococcus | 4 | 5.3% | 14.9% |
| Leuconostoc | 3 | 4.0% | 0.4% |
| Myceligenerans | 3 | 4.0% | 0.5% |
| Micrococcus | 3 | 4.0% | 0.5% |
| Ralstonia | 3 | 4.0% | 0.8% |
| Elizabethkingia | 3 | 4.0% | 0.8% |
| Pelomonas | 3 | 4.0% | 1.7% |
| Delftia | 3 | 4.0% | 2.8% |
| Sneathia | 3 | 4.0% | 0.9% |
| Pelistega | 3 | 4.0% | 6.4% |
| Bosea | 2 | 2.7% | 0.5% |
| Brevibacterium | 2 | 2.7% | 0.3% |
| Stenoxybacter | 2 | 2.7% | 0.3% |
| Paracoccus | 2 | 2.7% | 0.3% |
| Desulfobulbus | 2 | 2.7% | 0.3% |
| Simplicispira | 2 | 2.7% | 0.3% |
| Afipia | 2 | 2.7% | 0.3% |
| Sediminibacterium | 2 | 2.7% | 0.3% |
| Hyphomicrobium | 2 | 2.7% | 0.3% |
| Anoxybacillus | 2 | 2.7% | 0.3% |
| Cupriavidus | 2 | 2.7% | 0.3% |
| Alicycliphilus | 2 | 2.7% | 0.3% |
| Luteibacter | 2 | 2.7% | 4.3% |
| Deinococcus | 2 | 2.7% | 0.8% |
| Acidovorax | 2 | 2.7% | 0.8% |
| Chryseobacterium | 2 | 2.7% | 0.4% |
| Methylibium | 2 | 2.7% | 0.4% |
| Hydrogenophilus | 2 | 2.7% | 0.4% |
| Adhaeribacter | 2 | 2.7% | 0.4% |
| Dechloromonas | 2 | 2.7% | 0.4% |
| Pyramidobacter | 2 | 2.7% | 1.3% |
| Brevundimonas | 2 | 2.7% | 0.7% |
| Lactococcus | 2 | 2.7% | 0.9% |

"α": the proportion of TB patients in whom sequences from the corresponding genera were found

"β": the percentage of sequences of the corresponding genera of all sequences found in TB patients

**Table S3. OR and significance of existence of some specific microorganisms between TB patients and healthy controls.**

|  | TB | HC | X^2^ | P | Fisher's exact P |
| --- | --- | --- | --- | --- | --- |
| Haloplasma | 19 (25.3%) | 1 (5%) | 3.93 | 0.047 | 0.063 |
| Kocuria | 0 | 2 (10%) | 7.66 | 0.006 | 0.043 |
| Flavobacterium | 1 (1.3%) | 2 (10%) | 3.88 | 0.049 | 0.111 |
| Coprococcus | 1 (1.3%) | 4 (20%) | 11.03 | 0.001 | 0.007 |
| Clostridium | 4 (5.3%) | 4 (20%) | 4.40 | 0.036 | 0.058 |
| Acinetobacter | 9 (12%) | 6 (30%) | 3.85 | 0.050 | 0.079 |
| Pseudomonas | 18 (24%) | 11 (55%) | 7.15 | 0.007 | 0.013 |
| Moryella | 25 (33.3%) | 12 (60%) | 4.72 | 0.030 | 0.04 |
| Haemophilus | 21 (28%) | 12 (60%) | 7.13 | 0.008 | 0.016 |
| Catonella | 27 (36%) | 13 (65%) | 5.45 | 0.020 | 0.024 |
| Treponema | 25 (33.3%) | 14 (70%) | 8.77 | 0.003 | 0.005 |
| Oribacterium | 39 (52%) | 16 (80%) | 5.08 | 0.024 | 0.04 |
| Selenomonas | 35 (46.7%) | 18 (90%) | 12.02 | 0.001 | 0.001 |
| Leptotrichia | 56 (74.7%) | 19 (95%) | 3.93 | 0.047 | 0.035 |
| Fusobacterium | 60 (80%) | 20 (100%) | 4.75 | 0.029 | 0.035 |
| Porphyromonas | 56 (74.7) | 20 (100%) | 6.33 | 0.012 | 0.01 |

TB: Pulmonary tuberculosis patients

HC: Healthy controls

**Table S4. Distribution of the most abundant genus in each patient group.**

| Most abundant genus | N-TB (N=25) | R-TB (N=30) | F-TB (N=20) | HC (N=20) |
| --- | --- | --- | --- | --- |
| *Streptococcus* | 9 | 15 | 9 | 4 |
| *Prevotella* | 4 | 1 |  | 12 |
| *Neisseria* | 6 | 8 | 6 | 4 |
| *Veillonella* | 3 |  | 3 |  |
| *Alcaligenes* | 1 |  |  |  |
| *Lautropia* | 1 |  |  |  |
| *Leptotrichia* | 1 |  |  |  |
| *Stenotrophomonas* |  | 2 |  |  |
| *Rothia* |  | 1 |  |  |
| *Pseudomonas* |  | 1 | 1 |  |
| *Lactobacillus* |  | 1 |  |  |
| *Achromobacter* |  | 1 |  |  |
| *Granulicatella* |  | 0 | 1 |  |

**Figure S1. Rarefaction analysis of 16S rRNA gene sequences of 3 groups of TB patients compared with healthy controls.** F-TB, TB patients with treatment failure; HC, healthy controls; N-TB, new TB patients; R-TB, recurrent TB patients.


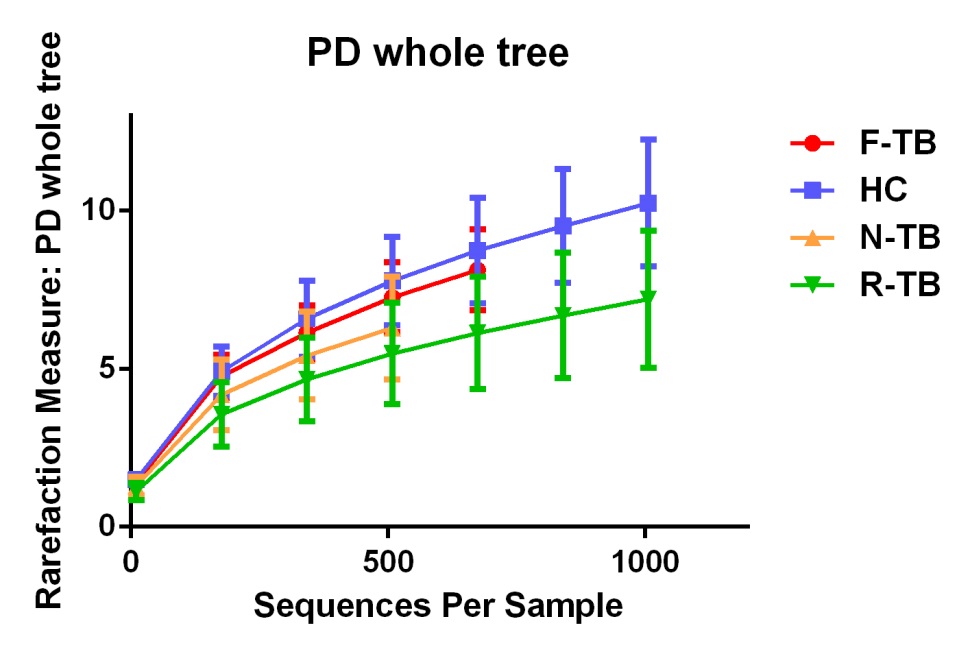


**Supplementary Information: detailed figure legend in Figure 3b.**


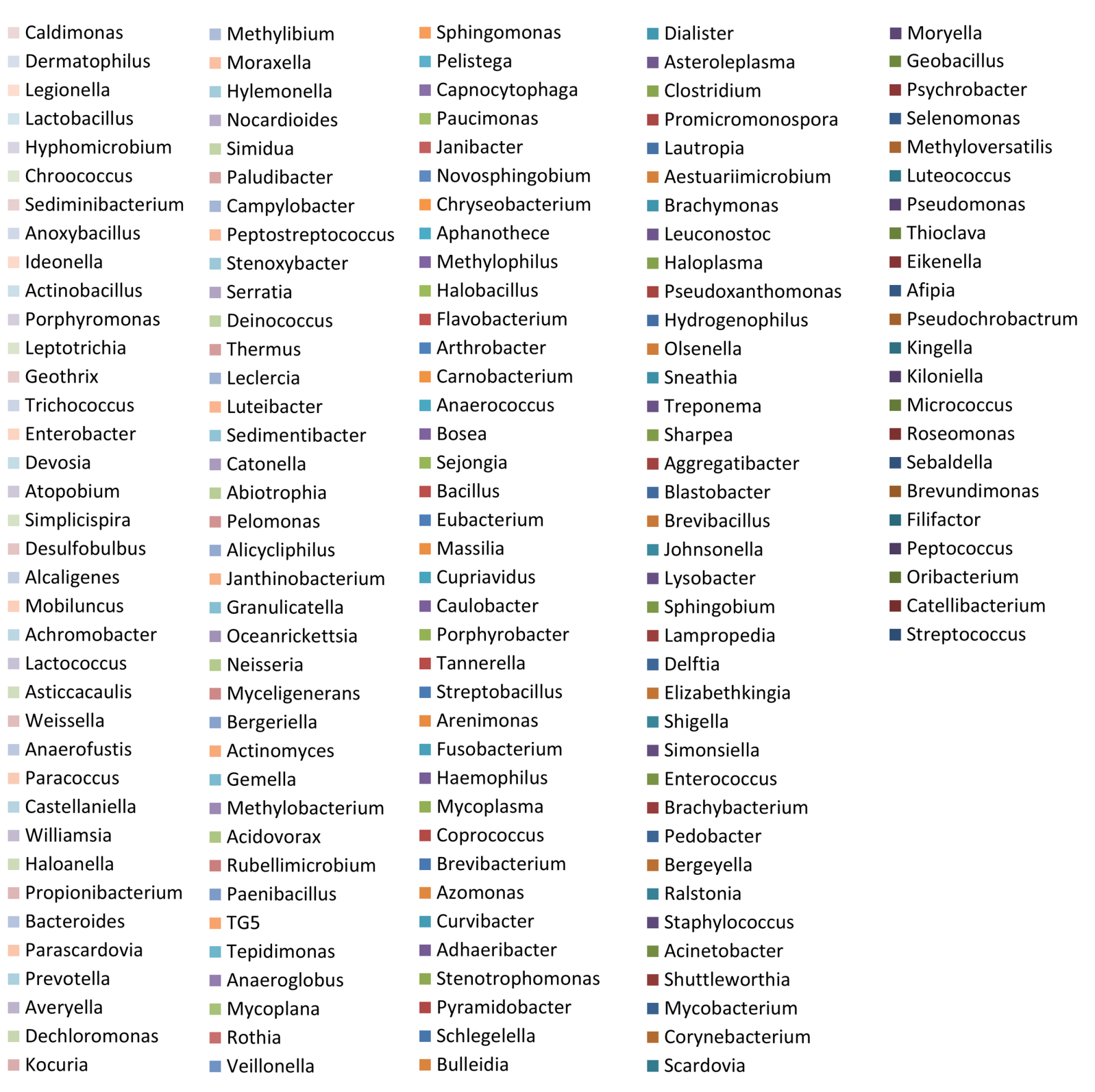

Supplement: File S1 — Figure S1, Rarefaction analysis of 16S rRNA gene sequences of 3 groups of TB patients compared with healthy controls. F-TB, TB patients with treatment failure; HC, healthy controls; N-TB, new TB patients; R-TB, recurrent TB patients. Table S1, List of barcoded primers used in the study. Table S2, The existence of genera which were uniquely found in the sputum of TB patients. Table S3, OR and significance of existence of some specific microorganisms between TB patients and healthy controls. Table S4, Distribution of the most abundant genus in each group. Information S1, detailed figure legend in Figure 3b. (DOC) [file pone.0083445.s001.doc]
